# Gene Expression Indicates Altered Immune Modulation and Signaling Pathway Activation in Ovarian Cancer Patients Resistant to Topotecan

**DOI:** 10.3390/ijms20112750

**Published:** 2019-06-05

**Authors:** Otília Menyhárt, János Tibor Fekete, Balázs Győrffy

**Affiliations:** 1 2nd Department of Pediatrics, Semmelweis University, Tűzoltó u. 7-9, H-1094 Budapest, Hungary; menyhart.Otilia@med.semmelweis-univ.hu (O.M.); jfeketet@hotmail.com (J.T.F.); 2 MTA TTK Lendület Cancer Biomarker Research Group, Institute of Enzymology, Hungarian Academy of Sciences, Magyar tudósok körútja 2., H-1117 Budapest, Hungary

**Keywords:** ovarian carcinoma, resistance, biomarker, gene expression, gene arrays, survival, tumor microenvironment

## Abstract

Epithelial ovarian cancer (EOC) is one of the deadliest gynecological malignancies. Topotecan remains an essential tool in second-line therapy; even so, most patients develop resistance within a short period of time. We aimed to identify biomarkers of topotecan resistance by using gene expression signatures derived from patient specimens at surgery and available subsequent responses to therapy. Gene expression was collected for 1436 patients and 10,103 genes. Based on disease progression, patients were categorized as responders/nonresponders depending on their progression free survival (PFS) state at 9, 12, 15 and 18 months after surgery. For each gene, the median expression was compared between responders and nonresponders for two treatment regimens (chemotherapy including/excluding topotecan) with Mann–Whitney U test at each of the four different PFS cutoffs. Statistical significance was accepted in the case of *p* < 0.05 with a fold change (FC) ≥ 1.44. Four genes (*EPB41L2*, *HLA-DQB1*, *LTF* and *SFRP1*) were consistently overexpressed across multiple PFS cutoff times in initial tumor samples of patients with disease progression following topotecan treatment. A common theme linked to topotecan resistance was altered immune modulation. Genes associated with disease progression after systemic chemotherapy emphasize the role of the initial organization of the tumor microenvironment in therapy resistance. Our results uncover biomarkers with potential utility for patient stratification.

## 1. Introduction

Ovarian cancer accounts for 2.5% of cancer diagnoses and 5% of cancer-related deaths among women worldwide. Despite gradually descending rates in some developed countries [1], incidence rates are on the rise in ageing societies [2]. At the same time, mortality rates have not improved significantly over the past decades. The most common subgroup, epithelial ovarian cancer (EOC), represents about 90% of all ovarian cancer cases. A lack of specific symptoms, late diagnoses and intrinsic and acquired resistance to most conventional chemotherapies designate EOC as one of the deadliest cancers among women, with 5-year survival rates as low as 50% [3]. Over 70% of patients are diagnosed in an advanced disease stage with intraperitoneal dissemination [4]. The most common subtype, serous ovarian carcinoma, represents about 50% of all EOC, with most diagnosed at stage III (51%) or stage IV (29%), with 5-year survival rates of 20%, reflecting the subtype’s aggressive nature [1]. 

Primary treatment consists of surgery and platinum-based combination chemotherapy, usually involving cisplatin [5,6]. High initial response rates are followed by frequent disease recurrence, and most original responders eventually develop resistance to conventional therapies [7,8]. Second-line treatment depends on the patient’s response to the first line and involves medicines such as topotecan, doxorubicin, paclitaxel, bevacizumab and gemcitabine [9,10,11,12]. Nevertheless, only 15–35% of patients respond to second-line therapy; thus, the development of efficacious salvage therapies is an unmet need, as is the identification of treatment-resistance biomarkers.

Topotecan is a semi-synthetic derivative of camptothecin that inhibits the activity of DNA topoisomerase I, which is required for DNA replication and transcription. Topotecan mimics a DNA base pair that binds to and stabilizes the enzyme–DNA complex, eventually leading to double-stranded DNA breaks and cellular death [13]. Topotecan was approved as a second-line therapy for relapsed EOC in 1996, with demonstrated antitumor activity in platinum-sensitive, platinum-resistant and paclitaxel-resistant tumors with a predictable and manageable toxicity profile [14], and to date it remains an important treatment option. Nonetheless, cancer cells develop various mechanisms to evade topotecan, such as by novel mutations or down-regulation of the topoisomerase gene [15]. Increased expression of collagen genes might also inhibit drug penetration to the tumor tissue [16], although their causative role in topotecan resistance has not been validated. The active removal of topotecan from cancer cells is also under intense scrutiny [17]. 

With most EOC patients developing resistance against systemic chemotherapy, biomarkers predicting specific treatment responses with potential utility for patient stratification for personalized therapeutic approaches are much sought after. We aimed to identify differentially expressed genes in patient specimens collected at surgery with an accessible response to systemic therapy, with particular focus on topotecan-containing regimens. Since topotecan is administered as the second line to a heavily pretreated population, patients not treated with topotecan may assist in revealing differential gene expression across treatment modalities. The initial gene expression in tumor specimens of subsequent nonresponders (defined as patients with disease progression until a certain cutoff time after surgery) allows the identification of upregulated genes associated with disease relapse.

Our findings suggest that cancer progression may be related to preceding alterations in the tumor microenvironment. Resistance associated with systemic chemotherapy seems to be linked to already present ECM and cytoskeleton modifications in initial tumor samples, while altered immune modulation is indicated in treatment-naïve specimens of patients who progressed on topotecan.

The presented bioinformatics-based concept helps to identify biomarkers of therapy resistance with potential utility for patient stratification, may be adapted for other treatments or tumor types, and provides a viable starting point for subsequent functional investigations. 

## 2. Results

### 2.1. Transcriptomic Database for Biomarker Selection

**Figure 1:**
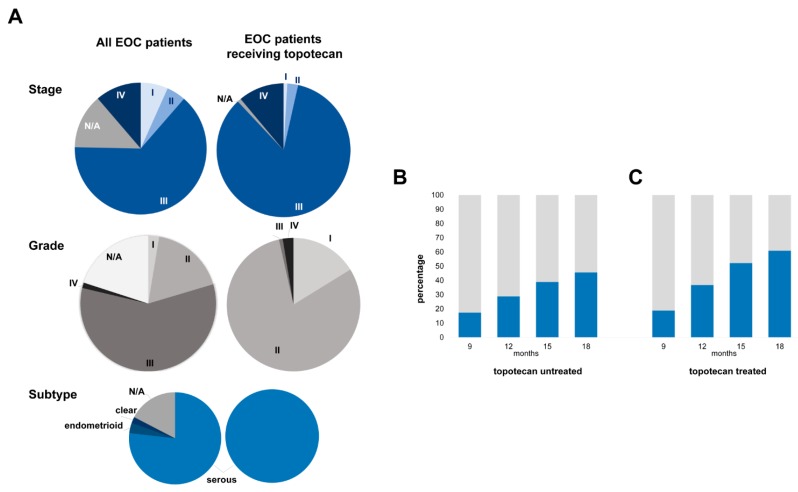
Patient characteristics including stage, grade and histological subtype across the entire epithelian ovarian cancer dataset and in the sub-cohort treated with systemic therapy including topotecan. (**A**) Proportion of responders (grey)/nonresponders (blue) to systemic chemotherapy including/excluding topotecan at the four progression free survival (PFS) cutoff times in topotecan-untreated (**B**) and -treated (**C**) patients.

Data from 1436 patients diagnosed with EOC were available from 10 datasets from the Gene Expression Omnibus (GEO) (https://www.ncbi.nlm.nih.gov/geo/) and The Cancer Genome Atlas (TCGA) (https://cancergenome.nih.gov/) repositories [18,19,20,21,22,23,24,25,26,27]. The majority (~77%) of EOC patients were diagnosed with serous subtype, and 64% of patients were identified with a stage III disease (Figure 1A). About half of patients had undergone optimal tumor debulking before chemotherapy initiation. The majority of patients (87.7%) received platinum-based chemotherapy, while about half of them received this in combination with taxol. Less than 10% of patients received either gemcitabine or topotecan, and only 3.5% of patients were treated with the targeted angiogenesis inhibitor bevacizumab. Over 26% of patients relapsed until the 12-month cutoff, and in total two-thirds of patients progressed during the median follow-up of 16.3 months (Table 1).

### 2.2. Upregulated Genes among Nonresponders Treated with Chemotherapy Excluding Topotecan

**Figure 2:**
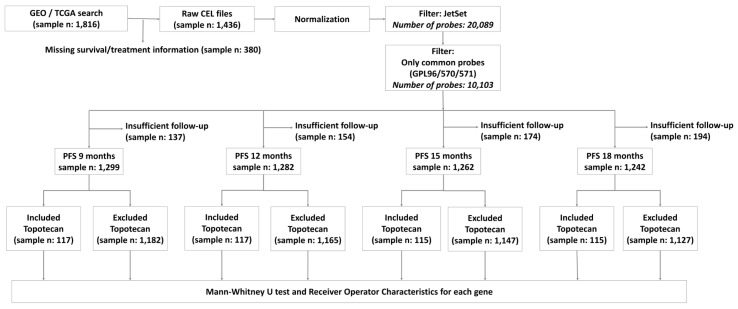
Analysis workflow for the database setup and the number of systemic chemotherapy-treated (including/excluding topotecan) patients at each PFS-cutoff.

Out of the available 1436 patients, 1319 were treated with systemic chemotherapy, not including topotecan. Some patients had to be excluded from subsequent analyses due to incomplete follow-up (Figure 2). Out of systemic chemotherapy-treated patients, 17.3% progressed until the 9th month and 45.6% until the 18th month of PFS cutoff after surgery (Figure 1B).

**Figure 3:**
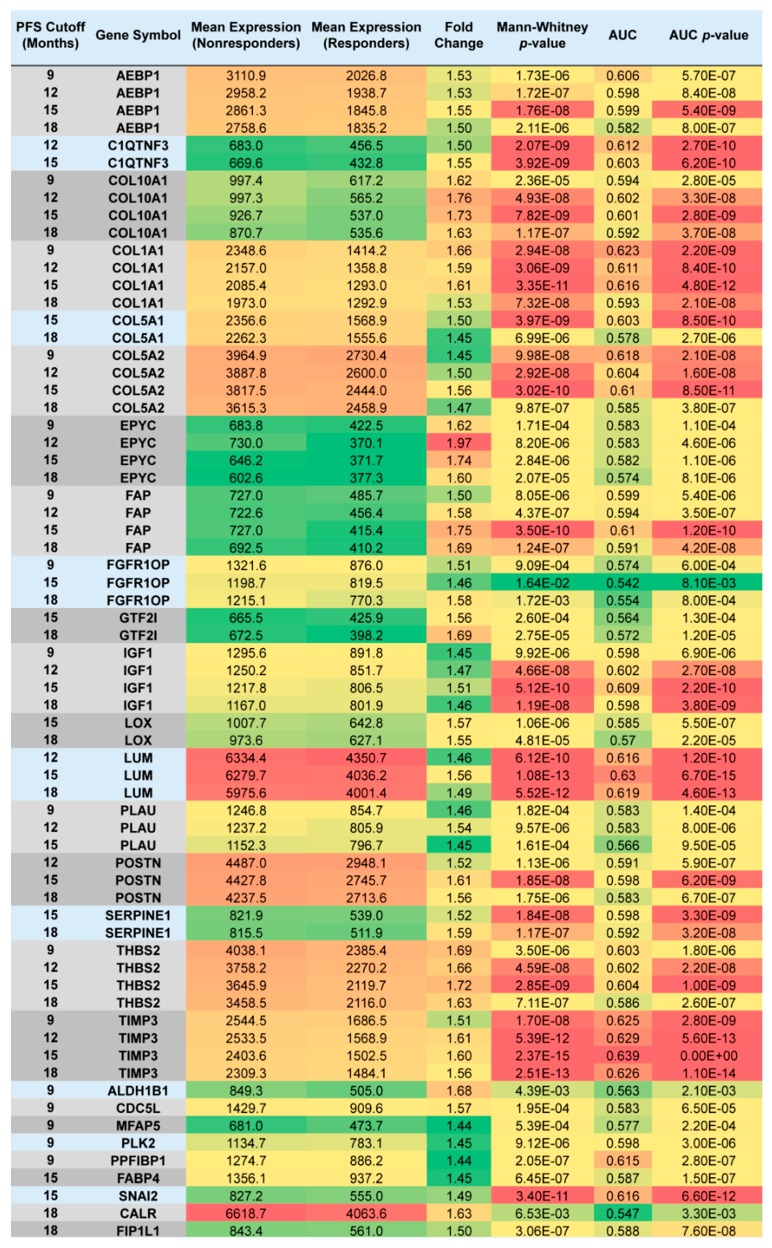
List of genes with higher expression in specimens of nonresponders who received systemic chemotherapy excluding topotecan. The figure is color-coded for expression (high-red), fold change (high-red) and *p*-values (low-red).

We compared the gene expression derived from initial tumor samples between subsequent responders and nonresponders at each of the four PFS cutoff points, and determined significantly upregulated genes in specimens of nonresponders (at *p* < 0.05; FC ≥ 1.44; mean expression in at least one cohort > 600). Altogether, 27 genes were overexpressed in tumor specimens of patients who progressed after systemic adjuvant therapy excluding topotecan until 18 months post-surgery. Out of the 27 genes, 16 were significantly upregulated in patients who relapsed until 9 months post-surgery, 13 genes were significant when PFS cutoff was set at 12 months, 20 genes when the cutoff was set at 15 months, and 18 genes when the cutoff time between nonresponders and responders was set at 18 months (Figure 3). 

For 18 genes, the association between gene upregulation and subsequent disease progression held through multiple cutoff times, and for 9 genes (*AEBP1*, *COL10A1*, *COL1A1*, *COL5A2*, *EPYC*, *FAP*, *IGF1*, *THBS2* and *TIMP3*), the association remained significant at all four PFS cutoffs between responders and nonresponders (Figure 3).

We subjected the 27 upregulated genes to the gene enrichment analysis by Database for Annotation, Visualization and Integrated Discovery (DAVID) Bioinformatics Resources 6.8. Gene sets related to extracellular matrix and collagen fibril organization, skeletal system development, cellular response to fibroblast growth factor stimulus and collagen catabolic process were enriched significantly (after Bonferroni correction) in tumor samples derived from patients who eventually became resistant to systemic chemotherapy excluding topotecan. The results, grouped by relevant function, are listed in Table 2.

### 2.3. Topotecan-Treated EOC Population

As topotecan is administered in second-line treatment, a more aggressive subtype and advanced stage, a heavy treatment load and poor survival outcome were expected in this patient cohort (*n* = 117). All topotecan-treated patients were diagnosed with serous EOC (Figure 1A). Consistent with this subtype’s aggressive nature, most patients (~85%) were recognized with a clinical stage III disease (Figure 1A). Three-quarters of patients underwent successful tumor debulking before starting on adjuvant systemic treatment. All topotecan-treated patients received platinum-based chemotherapy in the first line, and 75% of them obtained combined platinum/taxol treatment. Besides topotecan, the second-line treatments also involved gemcitabine (~62%), docetaxel (~31%), paclitaxel (~26%) and bevacizumab (~13%) (Table 1). During the median follow-up time of the entire cohort (14.8 months), 88% of topotecan-treated patients relapsed.

### 2.4. Upregulated Genes in Topotecan-Treated EOC Population

Out of the available 117 topotecan-treated patients, 18.8% were identified as non-responders by 9 months, and 36.7% of patients relapsed by 12 months after surgery. Survival data were available for 115 patients at 15 and 18-month cutoff times, out of which 52.1% of patients progressed until the 15th month and 60.9% until the 18-month PFS cutoffs (Figure 1C). We compared the expression of the 10,103 genes between specimens of responders and nonresponders determined at each PFS cutoff.

**Figure 4:**
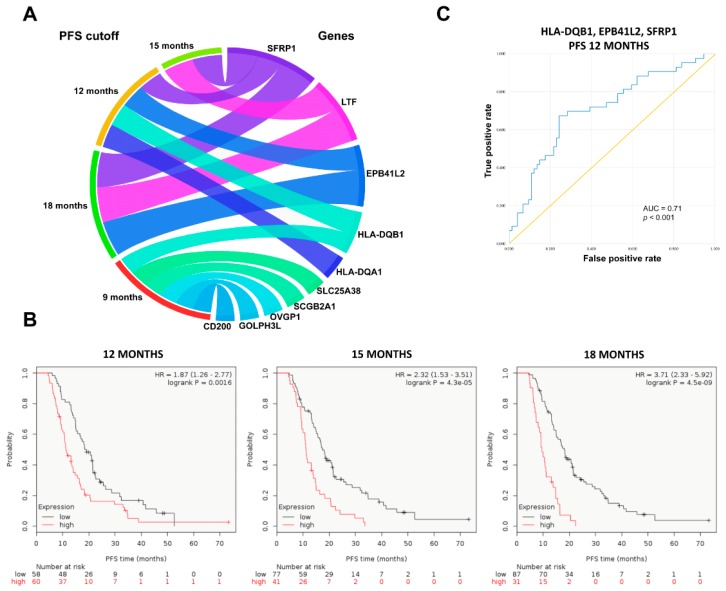
Significantly upregulated genes at the four different progression free survival (PFS) cutoff points in the topotecan-treated patient cohort (**A**). Combined expression of significant upregulated genes at 12, 15 and 18-month PFS cutoffs associated with disease progression in the group of topotecan-treated patients (**B**). Combined receiver operator characteristic (ROC) curve for *EPB41L2*, *HLA-DQB1* and *SFRP1*, consistently overexpressed in initial tumor samples of subsequent nonresponders to topotecan, which were also significant at the 12-month PFS cutoff (**C**).

In total, 10 upregulated genes were identified in nonresponders treated with topotecan-containing chemotherapy: six genes (*CD200*, *GOLPH3L*, *HLA-DQB1*, *OVGP1*, *SCGB2A1* and *SLC25A38*) were significantly upregulated in tumors of patients with subsequent disease progression when PFS cutoff was set at 9 months, four genes (*EPB41L2*, *HLA-DQA1*, *HLA-DQB1* and *SFRP1*) when the cutoff was at 12 months, two (*SFRP1* and *LTF*) genes at 15 months and three genes (*SFRP1*, *LTF* and *EPB41L2*) when the cutoff was at 18 months (Table 3, Figure 4A). 

The combined expression of genes upregulated at 12, 15 and 18-month cutoffs was associated with significantly worse PFS among topotecan-treated patients (Figure 4B), but the association was not significant for the combined gene expression at 9 months (*p* > 0.1).

**Figure 5:**
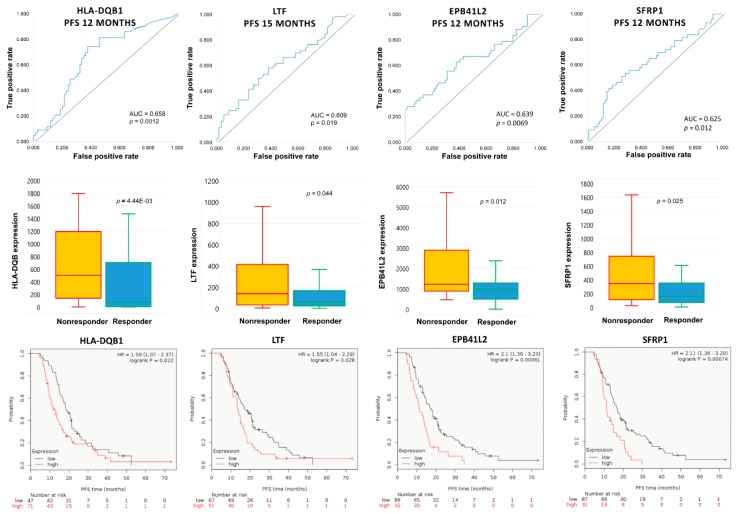
Consistently upregulated genes in EOC tumor specimens associated with subsequent resistance to topotecan-containing systemic chemotherapy. The high expression of the identified genes is associated with worse progression-free survival among topotecan-treated patients.

Four genes (*EPB41L2*, *HLA-DQB1*, *LTF* and *SFRP1*) were consistently overexpressed in initial tumor samples of subsequent nonresponders across multiple PFS cutoff times (Figure 5). High expressions of *EPB41L2*, *HLA-DQB1*, *LTF* and *SFRP1* were persistently associated with significantly worse PFS among topotecan-treated EOC patients (Figure 5). There was no correlation in the expression of *EPB41L2*, *HLA-DQB1*, *LTF* and *SFRP1* genes (Spearman’s rank correlation, *p* > 0.1). There was also no association between treatment response and different stages of EOC (χ^2^ test, *p* = 0.588).

Combined area under the curve (AUC) values for *EPB41L2*, *HLA-DQB1* and *SFRP1*, which were consistently overexpressed in initial tumor samples of subsequent nonresponders and significant at the 12-month cutoff, are presented in Table 4 and Figure 4C.

None of the ten upregulated genes identified in nonresponders treated with topotecan-containing chemotherapy overlapped to genes upregulated in nonresponders treated with systemic chemotherapy excluding topotecan.

## 3. Discussion

Our results support the significance of initial tumor microenvironment organization in subsequent therapy resistance. The tumor microenvironment is a heterogeneous cell population composed of stromal, tumor and immune cells and an extracellular matrix (ECM) [28], where tumor cells and the surrounding environment communicate substantially and their co-evolution promotes tumor growth and progression [29]. In relapsed patients treated with chemotherapy (excluding topotecan), upregulated genes were specific to the extracellular matrix and collagen fibril organization, skeletal system development, cellular response to fibroblast growth factor stimulus and collagen catabolic processes. 

Cancer-associated fibroblasts (CAF) are the most prominent stromal cell types that release a variety of factors into the tumor microenvironment that may promote ECM remodeling [30], and contribute to drug-resistance acquisition with a negative impact on clinical outcome [31]. A useful marker indicating the presence of CAFs, especially myofibroblasts, is the high intratumoral expression of fibroblast activation protein-α (FAP). FAP-expressing cells also exert an immune-suppressive function in the tumor microenvironment [32]. In our analysis, *FAP* was consistently overexpressed in treatment-naïve specimens of patients with subsequent relapse after cytotoxic chemotherapy, along with genes that contribute to EMT, angiogenesis, invasion and metastasis, conforming to previous findings [23,33,34]. Moreover, there was a compelling overlap between our list of upregulated genes in chemotherapy-resistant patients and the collagen-remodeling gene signature associated with poor outcomes in serous EOC [35]. 

Strikingly, a different set of genes was upregulated in initial samples of subsequent nonresponders to topotecan. Topotecan, with its well-tolerated toxicity profile, remains an important tool in the treatment of recurrent EOC, administered to an already pretreated population resistant to first-line chemotherapy. However, most patients eventually progress with limited options for salvage therapies. In our dataset, a high initial expression of *EPB41L2*, *HLA-DQB1*, *LTF* and *SFRP1* was linked to subsequent shorter progression-free survival. The overexpression of immune-function related genes, such as *HLA-DQA1*, *HLA-DQB1* and *LTF*, suggests the significance of immune modulation, while additional upregulated genes were linked to altered cell adhesion (*CD200*), Golgi to plasma membrane protein transport (*GOLPH3L*), androgen signaling (*SCGB2A1*), heme biosynthesis and erythrocyte differentiation (*SLC25A38*). 

These findings further reinforce the role of the altered tumor-microenvironment in subsequent therapy resistance. The spectrum of alterations present in treatment-naïve tumor samples affecting response to chemotherapy (ECM and cytoskeletal remodeling) or topotecan (immune modulation) highlight potential mechanisms of the evolution of cancer to a more aggressive form.

The class II HLA molecules of the human major histocompatibility complex (MHC) are expressed in macrophages, B-lymphocytes and dendritic cells, with a central role of presenting antigenic peptides derived from exogenous proteins to cognate CD4+ T-cells. HLA class II molecules are heterodimers and *HLA-DQA1* and *HLA-DQB1* encode members of the alpha and beta chain paralogs, respectively. During HLA molecule synthesis, class II α and β chains dimerize in the endoplasmic reticulum and form a nonameric complex with the invariant chain (Ii), which contributes to proper folding and prevents premature peptide loading [36]. The expression of HLA class II antigens is tightly regulated to ensure an adequate immune response towards pathogens, virally transformed and malignant cells [37]. 

Growing evidence indicates that the expression of HLA class II antigens by tumor cells alters their immunogenicity [38]. The expressed HLA class II molecules could make the tumor cells more detectable and eliminable for the immune system. Accordingly, constitutive HLA class II antigen expression is associated with a favorable prognosis in numerous solid tumors [39,40]. However, consistent with our results, HLA upregulation may be coupled with a lack of immune-mediated tumor eradication: constitutive HLA class II antigen expression has been associated with increased progression in melanomas [41,42] and linked to more frequent metastasis, recurrence, poor response to chemotherapy and dismal outcome in osteosarcoma [43]. In the ovarian carcinoma microenvironment, only T cells are able to spontaneously suppress tumor progression [44]. Consistent HLA class II overexpression in initial specimens of topotecan-treated and relapsed EOC patients suggests that HLA class II-mediated immune-escape deserves further consideration as a mechanism of therapy resistance.

Apart from antigen presentation, HLA class II components may have a role in tumorigenesis, as certain alleles or polymorphisms have been shown to contribute to cancer susceptibility [45]. A significant association has been established between the presence of HLA-class II haplotypes DRB1*0301-DQA1*0501-DQB1*0201 and DRB*1001-DQA1*0101-DQB1*0501 and increased risk of ovarian cancer [46]. In a sample of ten Caucasian ovarian cancer patients, chromosomal changes, especially gene or DNA amplifications were frequent in the HLA class II region, although copy number changes of 6p21.3 were inversely correlated to expression levels of HLA class II molecules [47]. 

An important component of the non-specific immune system is the protein product of lactotransferrin (*LTF*) that participates in first-line microbial host-defense and iron homeostasis [48]. LTF, also called lactoferrin, is an iron-binding glycoprotein found at the mucosal surface, also abundant in specific granules of neutrophils with antiviral, antimicrobial and antifugal properties [48]. Lactoferrin is up-regulated during inflammation; it activates the innate immune system by surface receptors generating LTF-containing immune complexes (LTF-IC) that trigger infiltration by monocytes and macrophages [49]. LTF-ICs are also able to switch anti-inflammatory M2 macrophages to an M1-like phenotype with pro-inflammatory properties [50]. Lactoferrin sequesters free iron and, by removing this essential substrate, fights bacterial growth [51] and contributes to the iron regulatory gene signature, utilized for the identification of high-risk breast cancer patients [52]. The consistent upregulation of *LTF* may suggest an inflamed tumor microenvironment, indicating the possibility of microbial infections and/or potentially altered iron metabolism that may contribute to subsequent resistance to systemic topotecan administration. LTF also has the capacity to inhibit proliferation and induce apoptosis in tumor cells [53]. Alternatively, since topotecan generally affects rapidly proliferating cancerous cells, one can speculate that tumors with increased *LTF* expression and reduced proliferation may show innate resistance to the effects of topotecan. 

Another potential tumor suppressor upregulated in topotecan-resistant patients is *SFRP1*, a member of the secreted frizzled-related protein (SFRP) gene family that shares sequence homology with Fzd receptors, can sequester Wnt ligands and antagonize Wnt signaling [54]. The tumor suppressor function of SFRPs varies between different SFRP members and different cancer types [55,56]. In high-grade serous ovarian carcinomas, SFRP1 protein loss has been described, and reduced expression was associated with promoter methylation [57], although the study did not investigate treatment effects. In rat bone marrow cells, SFRP1 expression was profoundly increased after a single dose of topotecan–oxaliplatin combination therapy [58]. In contrast, in our dataset, *SFRP1* mRNA was already high prior to topotecan treatment in subsequent nonresponders. The overexpression of SFRP1 has been described in basal-like breast cancer [59] and associated with the presence of lymph node metastases and decreased overall survival in gastric cancer [60]. Our findings suggest that *SFRP1* may act as an oncogene in topotecan-treated patients, or alternatively, tumor cells overexpressing *SFRP1* may be innately more resistant to the elimination of rapidly proliferating tumor cells. 

Erythrocyte membrane protein band 4.1 like 2 (*EPB41L2*) gene encodes the protein 4.1G, a member of the 4.1 superfamily of scaffold proteins with three additional paralogues. Contrary to the other paralogues, 4.1G is a prognostic biomarker of worse survival within our dataset of ovarian cancer patients (data not shown); this association was also confirmed by The Human Protein Atlas [61]. Thus, *EPB41L2* may behave as an oncogene in ovarian cancer, although the association requires further investigations. 

Our approach has several limitations. The presence of shared genes across various cutoff times may be attributed to similar evolutionary forces, but the results do not provide evidence for such mechanisms. Instead, we present a promising starting point for subsequent investigations focusing on underlying processes. The bottom line is the concept that could be adapted for other treatments or tumor types. Moreover, upregulated genes associated with progression at 9 months may be more relevant compared to genes linked to relapse at 18 months, but our analysis is unable to differentiate between them. Finally, there are some inconsistencies in the patterns of gene overexpressions. For example, *EPB41L2* is upregulated at 12 and 18 but not at 15 months, which is possibly caused by the relatively small sample size. As more data become available, we hope to correct such inconsistencies. 

In summary, our approach suggests directions to study treatment-dependent mechanisms of therapy resistance. The common pattern among the consistently overexpressed genes in topotecan-resistant patients is that many of them function as tumor suppressors in other types of solid tumors, drawing attention to the specific organization of signaling networks in ovarian cancer. The identified transcriptomic perturbations may assist patient stratifications and offer avenues for future studies to unveil mechanisms of topotecan resistance, with particular focus on the altered immune environment. 

## 4. Methods

### 4.1. Database Setup

We searched GEO (http://www.pubmed.com/geo) and TCGA (http://cancergenome.nih.gov) to identify datasets suitable for the analysis. In this, the keywords “ovarian”, “cancer”, “survival”, “GPL96”, “GPL570” and “GPL571” were used. The three geo platforms (GPL) refer to three Affymetrix gene array platforms which share identical probe sets to measure gene expression. Non-overlapping probe sets were not utilized in our analysis. Only publications with available raw microarray gene expression data, clinical treatment and response or survival information, and at least 20 patients were included.

The raw CEL files were MAS5 normalized in the R statistical environment (http://www.r-project.org) using the Affy Bioconductor library (Figure 2) [62]. A second scaling normalization was performed to set the mean expression on each chip to 1000 to reduce batch effects [63]. 

### 4.2. Clinical Data

Clinical data were collected manually for each sample. Each dataset was validated by at least two researchers (J.T.F. and B.G.) to ensure the reliable designation of clinical characteristics for each patient sample. We studied the association between initial gene expression and subsequent disease-progression in patients treated with systemic chemotherapy including/excluding topotecan. For this, we utilized the presence/absence of disease progression to split patients into two cohorts (nonresponders/responders) at each of the time points of 9, 12, 15 and 18 months after surgery for both (systemic chemotherapy including/excluding topotecan) treatment regimens. 

### 4.3. Gene Selecting Algorithm

Information about gene expression for the 1436 patients was available for 10,103 unique genes. For each gene, the median expression was compared between responders and non-responders for each treatment type (chemotherapy including/excluding topotecan) with Mann–Whitney U test and receiver operator characteristics (ROC) at each progression-free survival (PFS) cutoff (9, 12, 15 and 18 months after surgery). Statistical significance was accepted in the case of *p* < 0.05 and fold change (FC) ≥ 1.44. Only genes with a mean expression above 600 were considered to be meaningful. The package “roc” was used to calculate the area under the curve (AUC) and significance [64] (http://www.bioconductor.org).

For each cutoff time, we had to exclude a fraction of the patients from the analysis whose event-free follow up did not reach the cutoff (e.g., follow up was shorter than 12 months at the 12-month PFS cutoff and no progression occurred during this period). The final number of responders and nonresponders for each treatment type and cutoff is illustrated in Figure 2.

Finally, gene enrichment analysis was performed by the Database for Annotation, Visualization and Integrated Discovery (DAVID) Bioinformatics Resources 6.8 to assess the biological meaning of functionally related gene groups [65]. Bonferroni correction was applied to correct for multiple testing.

## Figures and Tables

**Table 1:** Clinical characterization of the entire epithelial ovarian cancer (EOC) dataset and the 117 patients receiving topotecan.

Characteristics		All EOC Patients (%)		Topotecan-Treated EOC Patients (%)
**Stage**				
	I	6.7		0.9
	II	4.7		2.6
	III	64.0		84.6
	IV	11.3		11.1
	N/A	13.4		0.9
				
**Grade**				
	I	2.6		16.2
	II	17.8		80.3
	III	58.3		0.9
	IV	1.3		2.6
	N/A	20.0		
				
**Subtype**				
	Serous	76.9		100
	Endometrioid	3.6		
	Clear	2.2		
	N/A	17.4		
				
**Debulking**				
	Optimal	48.5		74.4
	Suboptimal	32.0		22.2
	N/A	19.5		3.4
				
				
**Progression**	(median follow up 16.3 months)	68.1	(median follow up 14.8 months)	88.0
				
**Treatment**				
	Platinum	87.7		100
	Taxol	49.8		
	Combined platinum and taxol	48.6		75.2
	Bevacizumab	3.5		12.8
	Docetaxel	7.4		30.8
	Gemcitabine	9.1		62.4
	Paclitaxel	15.9		25.6
	Topotecan	8.2		100

**Table 2:** Functional grouping of significantly upregulated genes in tumor samples of patients with subsequent resistance to systemic chemotherapy, excluding topotecan. Gene ontology analysis is based on 27 genes for which the association between gene upregulation and subsequent disease progression held through different cutoff times.

Term	Count	*p*-Value	Genes	Bonferroni
Extracellular matrix organization	9	3.90 × 10^−10^	*COL5A1*, *COL1A1*, *LOX*, *POSTN*, *COL5A2*, *COL10A1*, *LUM*, *SERPINE1*, *MFAP5*	1.31 × 10^−7^
Collagen fibril organization	5	3.58 × 10^−7^	*COL5A1*, *COL1A1*, *LOX*, *COL5A2*, *LUM*	1.20 × 10^−4^
Skeletal system development	6	1.93 × 10^−6^	*IGF1*, *COL1A1*, *POSTN*, *COL5A2*, *COL10A1*, *AEBP1*	6.45 × 10^−4^
Cellular response to fibroblast growth factor stimulus	4	1.30 × 10^−5^	*COL1A1*, *POSTN*, *SNAI2*, *CDC5L*	4.35 × 10^−3^
Collagen catabolic process	4	1.29 × 10^−4^	*COL5A1*, *COL1A1*, *COL5A2*, *COL10A1*	4.23 × 10^−2^

**Table 3:** Significantly upregulated genes in tumor samples of subsequent nonresponders identified at different cutoff times (9, 12, 15 and 18 months after surgery) treated with systemic chemotherapy including topotecan. The four highlighted genes were significantly upregulated in tumor samples of subsequent nonresponders across multiple PFS cutoff times. AUC: area under the curve.

PFS Cutoff (Months)	Gene Symbol	Mean Expression (Nonresponder)	Mean Expression (Responder)	Fold Change	Mann–Whitney *p*-Value	AUC	AUC *p*-Value
9	*CD200*	1718.6	1165.7	1.47	2.13 × 10^−2^	0.658	8.20 × 10^−3^
12	*EPB41L2*	1918.2	1160.9	1.65	1.24 × 10^−2^	0.639	6.90 × 10^−3^
18	*EPB41L2*	1703.6	1036.1	1.64	7.70 × 10^−3^	0.648	1.80 × 10^−3^
9	*GOLPH3L*	2289.1	1583.0	1.45	2.34 × 10^−2^	0.656	6.50 × 10^−3^
12	*HLA-DQA1*	1353.5	908.2	1.49	1.94 × 10^−2^	0.63	8.30 × 10^−3^
9	*HLA-DQB1*	1243.8	830.4	1.50	2.42 × 10^−2^	0.655	8.20 × 10^−3^
12	*HLA-DQB1*	1202.6	737.0	1.63	4.45 × 10^−3^	0.658	1.20 × 10^−3^
15	*LTF*	1034.3	316.5	3.27	4.38 × 10^−2^	0.609	1.90 × 10^−2^
18	*LTF*	908.5	352.8	2.58	4.13 × 10^−2^	0.613	1.70 × 10^−2^
9	*OVGP1*	1975.9	362.2	5.46	7.95 × 10^−3^	0.682	5.90 × 10^−3^
9	*SCGB2A1*	11433.0	7001.9	1.63	3.31 × 10^−2^	0.646	2.00 × 10^−2^
12	*SFRP1*	882.5	401.5	2.20	2.46 × 10^−2^	0.625	1.20 × 10^−2^
15	*SFRP1*	727.7	393.8	1.85	1.49 × 10^−2^	0.632	5.80 × 10^−3^
18	*SFRP1*	679.6	394.3	1.72	3.23 × 10^−2^	0.619	1.40 × 10^−2^
9	*SLC25A38*	1838.8	1101.3	1.67	2.55 × 10^−2^	0.707	1.90 × 10^−4^

**Table 4:** Combined AUC values involving three genes (*EPB41L2*, *HLA-DQB1* and *SFRP1*) which were consistently overexpressed in initial tumor samples of subsequent nonresponders to topotecan and also significant at the 12-month PFS cutoffs.

Gene Combinations	AUC Values	*p*-Values
*HLA-DQB1 + EPB41L2*	0.679	0.001
*HLA-DQB1 + SFRP1*	0.696	<0.001
*EPB41L2 + SFRP1*	0.675	0.002
*HLA-DQB1 + EPB41L2 + SFRP1*	0.71	<0.001
